# Radiation therapy affects YAP expression and intracellular localization by modulating lamin A/C levels in breast cancer

**DOI:** 10.3389/fbioe.2022.969004

**Published:** 2022-08-24

**Authors:** Giuseppe La Verde, Valeria Artiola, Mariagabriella Pugliese, Marco La Commara, Cecilia Arrichiello, Paolo Muto, Paolo A. Netti, Sabato Fusco, Valeria Panzetta

**Affiliations:** ^1^ Istituto Nazionale di Fisica Nucleare, INFN Sezione di Napoli, Naples, Italy; ^2^ Dipartimento di Farmacia, Università Degli Studi di Napoli Federico II, Naples, Italy; ^3^ Dipartimento di Fisica “Ettore Pancini”, Università Degli Studi di Napoli Federico II, Naples, Italy; ^4^ Radiotherapy Unit, Istituto Nazionale Tumori-IRCCS-Fondazione “G. Pascale”, Naples, Italy; ^5^ Interdisciplinary Research Centre on Biomaterials (CRIB) and Dipartimento di Ingegneria Chimica, Dei Materiali e Della Produzione Industriale, Università Degli Studi di Napoli Federico II, Naples, Italy; ^6^ Center for Advanced Biomaterials for Healthcare@CRIB, Istituto Italiano di Tecnologia, Naples, Italy; ^7^ Department of Medicine and Health Sciences “V. Tiberio”, University of Molise, Campobasso, Italy

**Keywords:** breast cancer, mechanobiology, extracellular matrix stiffness, YAP, lamin A/C, radiotherapy

## Abstract

The microenvironment of breast cancer actively participates in tumorigenesis and cancer progression. The changes observed in the architecture of the extracellular matrix initiate an oncogene-mediated cell reprogramming, that leads to a massive triggering of YAP nuclear entry, and, therefore, to cancer cell proliferation, invasion and probably to increased radiation-resistance. However, it is not yet fully understood how radiotherapy regulates the expression and subcellular localization of YAP in breast cancer cells experiencing different microenvironmental stiffnesses. To elucidate the role of extracellular matrix stiffness and ionizing radiations on YAP regulation, we explored the behaviour of two different mammary cell lines, a normal epithelial cell line (MCF10A) and a highly aggressive and invasive adenocarcinoma cell line (MDA-MB-231) interacting with polyacrylamide substrates mimicking the mechanics of both normal and tumour tissues (∼1 and ∼13 kPa). We report that X-ray radiation affected in a significant way the levels of YAP expression, density, and localization in both cell lines. After 24 h, MCF10A and MDA-MB-231 increased the expression level of YAP in both nucleus and cytoplasm in a dose dependent manner and particularly on the stiffer substrates. After 72 h, MCF10A reduced mostly the YAP expression in the cytoplasm, whereas it remained high in the nucleus of cells on stiffer substrates. Tumour cells continued to exhibit higher levels of YAP expression, especially in the cytoplasmic compartment, as indicated by the reduction of nuclear/cytoplasmic ratio of total YAP. Then, we investigated the existence of a correlation between YAP localization and the expression of the nuclear envelope protein lamin A/C, considering its key role in modulating nuclear deformability and changes in YAP shuttling phenomena. As supposed, we found that the effects of radiation on YAP nucleus/cytoplasmic expression ratio, increasing in healthy cells and decreasing in tumour ones, were accompanied by lower and higher lamin A/C levels in MCF10A and MDA-MB-231 cells, respectively. These findings point to obtain a deeper knowledge of the role of the extracellular matrix and the effects of X-rays on YAP and lamin A/C expression that can be used in the design of doses and timing of radiation therapy.

## 1 Introduction

Breast cancer is one of the most diagnosed diseases in women (Ferlay et al., 2018; DeSantis et al., 2019), which incidence increases together with age and other factors, such as ethnicity and family history of cancer (Coughlin, 2019). Therefore, together with prevention, enhancement and optimization of conventional treatments are fundamental for the reduction of its mortality. From several decades one of the most widely used treatment for breast tumours is radiotherapy since that the X-rays, produced by the linear accelerator (LINAC), can severely damage the DNA of the cells, through the formation of double-stranded breaks (Iliakis et al., 2003). The effect of ionizing radiations on the cell is well known in the literature: many studies have proven how radiation can provoke almost half of the DNA lesions leading to a plethora of consequences, such as carcinogenesis, cell death, or mutation (Elkind, 1984; Sinclair and Fry, 1987; Smith, 1987; Ward, 1988). On the other hand, yet a small number of investigations have focused on the mechanobiology of irradiated cell and tissues. It is nowadays well established a direct connection between the development of cancer and the alteration in the components of the cytoskeleton (CSK) (Hall, 2009; Panzetta et al., 2017), a structure that regulates several biological processes (Krieg et al., 2019; Ladoux and Mège, 2017). Specifically, during cancer transformation, the CSK is subjected to modifications in its arrangement and composition, generally accompanied by a lowering of the cell mechanical properties (Yilmaz and Christofori, 2009; La Verde et al., 2021). The reorganization of the CSK in tumour cells may results in epithelial-mesenchymal transition (EMT), which can promote cell migration and tumour invasiveness. Another biological structure essential to the correct functioning of cells and tissues is the extracellular matrix (ECM), which, in the transformation process of a healthy tissue into a tumoral one, stiffens, increasing its mechanical properties (Panzetta et al., 2017). In this regard, a massive effort is underway to elucidate the precise relationship existing between ECM mechanics and cell oncogenic reprogramming. And, even if not everything has been understood, a growing body of evidence indicates that the ECM stiffening (typical of ageing, inflammation, fibrosis, diabetes and smoking) (Panciera et al., 2017) can instruct normal cells to undergo a profound reprogramming and to acquire a tumour malignant phenotype. It has been demonstrated, in fact, that matrices recapitulating the stiffnesses of fibrotic tissues can promote some elements of this process, by inducing changes in cell shape, reduction in E-cadherin, followed by increase of N-cadherin, nuclear localization of β-catenin (Wei et al., 2015; Fattet et al., 2020), an increase in cell proliferation and a more active invasion process (Panzetta et al., 2017; Stowers et al., 2017; Panciera et al., 2020), particularly for breast cancer (Li et al., 2008; Baker et al., 2010; Nikkhah et al., 2010; Plodinec et al., 2012). Taken together these facts demonstrate how the loss of tissue homeostasis and diseases onset are strictly correlated to the point that some traditional and novel cancer treatments are targeting these structures (Karahalil et al., 2019). Indeed, going deeper, another fundamental function of the CSK is the conversion of mechanical signal into biochemical responses. With the mechanotransduction process, the CSK can pick mechanical stimuli and send them to the cell through the activation of mechanosensors, like Yes-associated protein (YAP)/Transcriptional coactivator with PDZ-binding motif (TAZ) complex (Low et al., 2014a). YAP is a transcriptional coactivators protein that, together with TAZ is strictly associated to mechanical and structural changes in the cell microenvironment. These proteins can move from the cytoplasm to the nucleus, where they interact with the TEA domain (TEAD) (Piccolo et al., 2014), association considered fundamental to promote their transcriptional abilities (Zhao et al., 2008; Chan et al., 2009; Zanconato et al., 2015). In healthy tissues, YAP moves from the nucleus to the cytoplasm (Dupont et al., 2011), where they can be degraded or inactivated, whereas in tumoral tissues YAP moves in the other direction where its transcriptional activity can be activated (Nukuda et al., 2015; Pocaterra et al., 2020). Additionally, it was reported that these proteins are usually stimulated during the development of most solid tumours, inducing cell proliferation, and increasing cells’ ability to create metastases (Camargo et al., 2007; Dong et al., 2007; Zhao et al., 2007; Chan et al., 2008; Zanconato et al., 2016a). YAP/TAZ complex is becoming a target in some cancer therapies since it has been proved that there is an increased expression of YAP and TAZ in the cell’s nucleus in KRAS-mutated cells, such as the invasive adenocarcinoma cell line MDA-MB-231. Conversely, the normal epithelial cell line MCF10A shows high concentrations of YAP in the cytoplasm (Panciera et al., 2020). Some recent studies have also reported a direct correlation between YAP and cell resistance to radiation. To high levels of YAP activation is associated a low response to X-rays, while YAP silencing increases sensitivity to radiation and the cell DNA damage (Fernandez-L et al., 2012; Akervall et al., 2014; Xu et al., 2019). Thus, all this indicates the necessity to implement new therapeutical approaches that consider the different and complex mechanisms underlying tumoral treatment. In this frame, we here investigated how the combination of different X-ray doses and ECM stiffness regulates the expression of YAP in two different mammary cell lines. The healthy cell line, MCF10A, and its tumoral counterpart, MDA-MB-231 were seeded on type I collagen functionalized polyacrylamide substrates, characterised by a Young’s Modulus of 1.3 and 13 kPa to recapitulate some characteristics of the healthy and cancerous tissue respectively. In fact, breast cancer with its characteristic highly fibrotic collagen content shows an increased stiffness (5–10 kPa) in comparison with healthy breast tissue characterized by a stiffness of 1 kPa (Levental et al., 2009; Plodinec et al., 2012). Once interacting with mechanically different substrates, cells were exposed to two doses of X-rays: 2 and 10 Gy, corresponding the former to the daily dose delivered in conventional radiotherapy and the latter to the maximum dose employed in metastases treatment. Specifically, here we report a first attempt to study the role that the substrate stiffness plays in mediating the cellular response to X-ray radiation in terms of YAP expression, density, and localization. Then, we investigated the existence of a correlation between YAP localization and the expression of the nuclear envelope protein lamin A/C, considering its key role in modulating nuclear deformability and changes in YAP shuttling phenomena. Importantly, we found that X-ray radiation affected YAP localization, increased in nuclei of healthy cells, and decreased in those of tumour ones, concurrently with the reduction and the enhancement of lamin A/C levels in MCF10A and MDA-MB-231 cells. These findings underscore the necessity to further examine the effects that X-rays induce on YAP and lamin A/C expression, in relation to the mechanical microenvironment, on subsequent cell behaviour (i.e., radiation sensitization or induction of radiation resistance). Such knowledge could be useful in tailoring therapeutic procedures and especially in the design of doses and timing of radiation therapy.

## 2 Materials and methods

### 2.1 Polyacrylamide substrate preparation

Polyacrylamide substrates were prepared and functionalized as previously reported (Panzetta et al., 2020). Specifically, 2 different formulations were prepared: 4% acrylamide/0.15% methylene-bis-acrylamide and 10% acrylamide/0.1% methylene-bis-acrylamide corresponding to 1.3 and 13 kPa (Young’s modulus), respectively. The substrates were functionalized with a solution of bovine type I collagen (50 μg/ml) using a bifunctional photoreactive crosslinker (sulfosuccinimidyl 6-(4′-azido-2′-nitrophenylamino) hexanoate, sulfo-SANPAH; Fischer Scientific, Loughborough, United Kingdom). Mechanical measurements substrates were performed by a stress-controlled shear rheometer (Anton Paar MCR 502) equipped with 25 mm stainless steel parallel plate geometry tool and a Peltier heating system to control the temperature at 37°C. Dynamic frequency sweeps were performed with frequency ranging from 10^–2^ to 10 Hz in the linear regime (strain of 0.1%, Supplementary Figure S1).

### 2.2 Cell culture

The cell lines analysed in this study were the healthy MCF10A cell line, and the triple-negative cancerous one, MDA-MB-231. The former was cultured in Lonza Dulbecco’s Modified Eagle Medium (DMEM/F-12) supplemented with 0.4% Bovine Pituitary Extract (BPE), 0.1% Human Epidermal Growth Factor (hEGF), 0.1% insulin, 0.1% hydrocortisone, 1% penicillin-streptomycin. MDA-MB-231 cells were cultured in the same basal medium supplemented with 10% foetal bovine serum (FBS), 1% L-Glutamine, and 1% penicillin-streptomycin. ∼10^6^ cells were seeded per polyacrylamide substrates (∼12.5 ⋅ 10^3^ cells/cm^2^), obtaining the cell confluence condition.

### 2.3 X-ray irradiation

Cells were irradiated using the Synergy Agility LINAC produced by ELEKTA company, characterised by a field size of 20 × 20 cm^2^. The samples were irradiated at the National Cancer Institute “Pascale” of Naples with a 6 MV photon beam, usually employed in the conventional treatment. The cell plates were placed between two plexiglass plaques, the one on top thinner than the other, to attenuate the radiations and emulate the skin sparing effect.

### 2.4 Immunofluorescence

To analyse the samples, 24 and 72 h after irradiation, cells were fixed using 4% paraformaldehyde, heated to 37 °C, for 15 min. Afterwards, the samples were washed with Phosphate Buffered Saline (PBS). The immunofluorescence procedure can be divided into three phases: permeabilization, blocking, and immunostaining. For the permeabilization process, cell plates were covered with 250 μl of Triton-X 100, diluted at 0.1%, for 10 min. Afterwards, for the blocking phase, the samples were incubated with 250 µl of Bovine Serum Albumin (BSA) at 1% for 1 h at room temperature. Then, lamin A/C was localized by mouse monoclonal lamin A/C antibody (Santacruz, SC-376248) and Alexa488 goat anti-mouse secondary antibodies (Life Technologies, A11008). YAP was localized by YAP1 polyclonal rabbit antibody (PA1-46189, ThermoFisher Scientific) and Alexa546 mouse anti-rabbit secondary antibody.

### 2.5 Confocal acquisition

To quantify YAP concentration and lamin A/C level in cells, the samples were observed with Olympus confocal microscope with a 63× objective. 10 z-stack images (12-bit color), averaging 4 frames each acquisition, were acquired for each sample. Each image was characterized by a size of 13.8 μm × 13.8 μm with a pixel size of 0.13 μm.

### 2.6 YAP analysis

Total YAP expression in both cell’s nucleus, $Y_{N}$ , and cytoplasm, $Y_{C}$ , was investigated employing ImageJ Fiji software (NIH, Bethesda, MD, United States ). Briefly, the z-stacks for the red channel (YAP) were projected into a single image using the “sum projection” function in ImageJ. YAP and lamin A/C images were used to extract individual cellular and nuclear outlines using ImageJ ROI manager tool and YAP expression at each condition was evaluated in terms of integrated fluorescence intensity within individual cellular and nuclear boundaries, $Y_{Cell}$ and $Y_{N}$ , respectively. The total YAP expression in the cytoplasm was calculated as difference between $Y_{Cell}$ and $Y_{N}$ . Then, the following parameters were evaluated:

$$Y_{N/C}=\;\frac{Y_{N}}{Y_{C}}$$ (1)

representing nuclear to cytoplasmic ratio of total YAP. Values lower or higher than 1 indicate prevalent localization of YAP in the cytoplasm or the nucleus, respectively.

$$Y_{N}^{d}=\;\frac{Y_{N}}{A_{N}}$$ (2)

$$Y_{C}^{d}=\;\frac{Y_{C}}{A_{Cell}-A_{N}}$$ (3)

where $A_{N}$ and $A_{C}$ are the nucleus and the cytoplasm area, whereas $Y_{N}^{d}$ and $Y_{C}^{d}$ represent the nuclear and cytoplasmic density/concentration of YAP, respectively.

Finally, the nuclear to cytoplasmic ratio of YAP density was calculated:

$$Y_{N/C}^{d}=\;\frac{Y_{N}^{d}}{Y_{C}^{d}}$$ (4)

This parameter is the most used to study the effects of translocation processes from nucleus to cytoplasm and vice versa and indicates if YAP is more concentrated into the cytoplasm ( $Y_{N/C}^{d}\ll 1$ ) or in the nucleus ( $Y_{N/C}^{d}\gg 1$ ).

All the analyses were carried out for both cell lines, doses, and times.

Considering that the analysis of the YAP fluorescence from the slices on the top and on the bottom of the nucleus may give a signal classified as belonging to the nucleus instead of to the cytoplasmic compartment, the analysis of all the parameters above introduced was performed by following a different procedure for a set of randomly selected cells in different conditions (13 cells). For the analysis of YAP in the nucleus, the slices where the nucleus is present were extracted and projected into a single image using again the ‘sum projection’ function in ImageJ. Then, $Y_{N}$ was evaluated in terms of integrated fluorescence intensity within the nuclear boundaries and used as real YAP expression in the nucleus ( $Y_{N}^{R}$ **)**. The analysis of all the other parameters was performed as previously described ( $Y_{C}^{R}$ and $Y_{N/C}^{R}$ ). The error committed for $Y_{N}$ , $Y_{C}$ and $Y_{N/C}$ was evaluated as: $\varepsilon \%=\frac{Y-Y^{R}}{Y}\%$ (Supplementary Figure S2).

### 2.6 Lamin A/C analysis

To quantify lamin A/C level, the z-stacks for the green channel (lamin A/C) were projected into a single image using the “maximum projection” function in ImageJ. Then, lamin A/C expression at each condition was evaluated in terms of integrated fluorescence intensity within individual nuclear boundaries.

### 2.7 Statistical analysis

Statistical comparisons were performed with a nonparametric Kruskal-Wallis test followed by Dunn-Bonferroni post-hoc method with *p*-values < 0.05 considered statistically significant.

## 3 Results and discussion

### 3.1 Radiation effects on nuclear to cytoplasmic YAP ratio density

The Hippo-YAP/TAZ pathway is an evolutionary conserved mechano-signalling pathway that has a crucial role in regulating organ size and tumorigenesis by moderating the balance between cellular proliferation and apoptosis. Inhibition of the Hippo-YAP/TAZ signalling pathway promotes the translocation of YAP/TAZ into the nucleus, thereby allowing the activation of the downstream genes. It has also been demonstrated that overexpression of YAP enhances tumorigenesis and metastasis also *in vivo* by inducing the EMT process and then, the upregulation of N-cadherin followed by the downregulation of E-cadherin. Furthermore, the role of YAP in mediating radiotherapy and chemotherapy resistance has been the subject of many studies that have indicated that high levels of YAP expression correlate with poor cell response to radiation therapy (Fernandez-L et al., 2012; Akervall et al., 2014). Further, YAP nuclear expression levels were demonstrated be correlated with poor prognosis of patients and with low sensitivity to radiation (Tsujiura et al., 2014).

**FIGURE 1:**
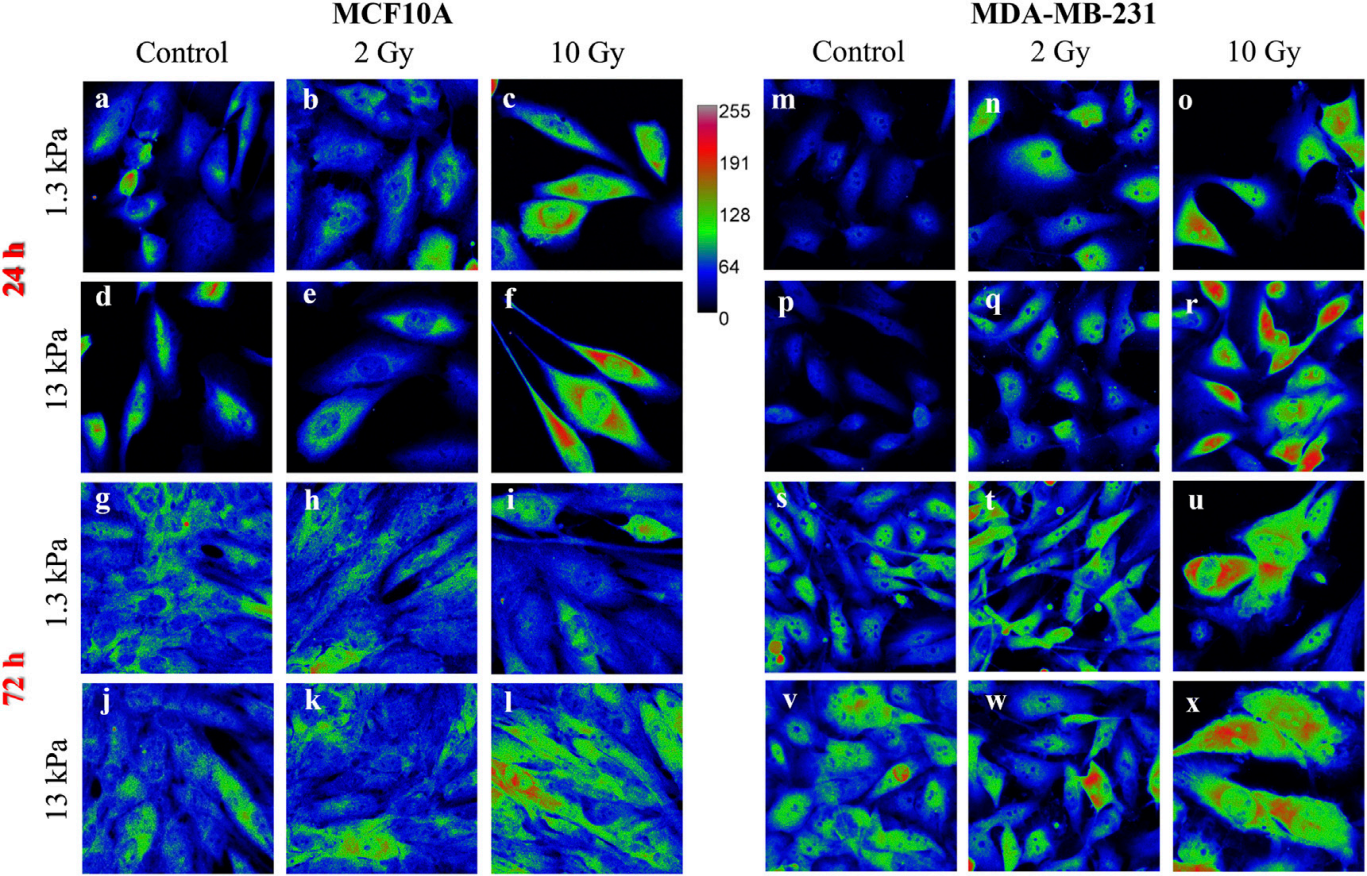
Sum intensity projections of z-stack images taken from YAP immunofluorescence in MCF10A **(A–L)** and MDA-MB-231 **(M–X)**, shown as rainbow RGB look-up table. Colour bar: YAP intensity **(A–U)**. Scale bar, 50 μm.

Nevertheless, little is known about the effects of radiation on YAP expression and localization in breast cells interacting with physio-pathological microenvironments. In particular, the effects of radiation on the localization of YAP were evaluated using Eq. 4, where YAP concentration of both the nucleus and the cytoplasm was calculated measuring the integrated fluorescence with the ImageJ software (Figure 1).

**FIGURE 2:**
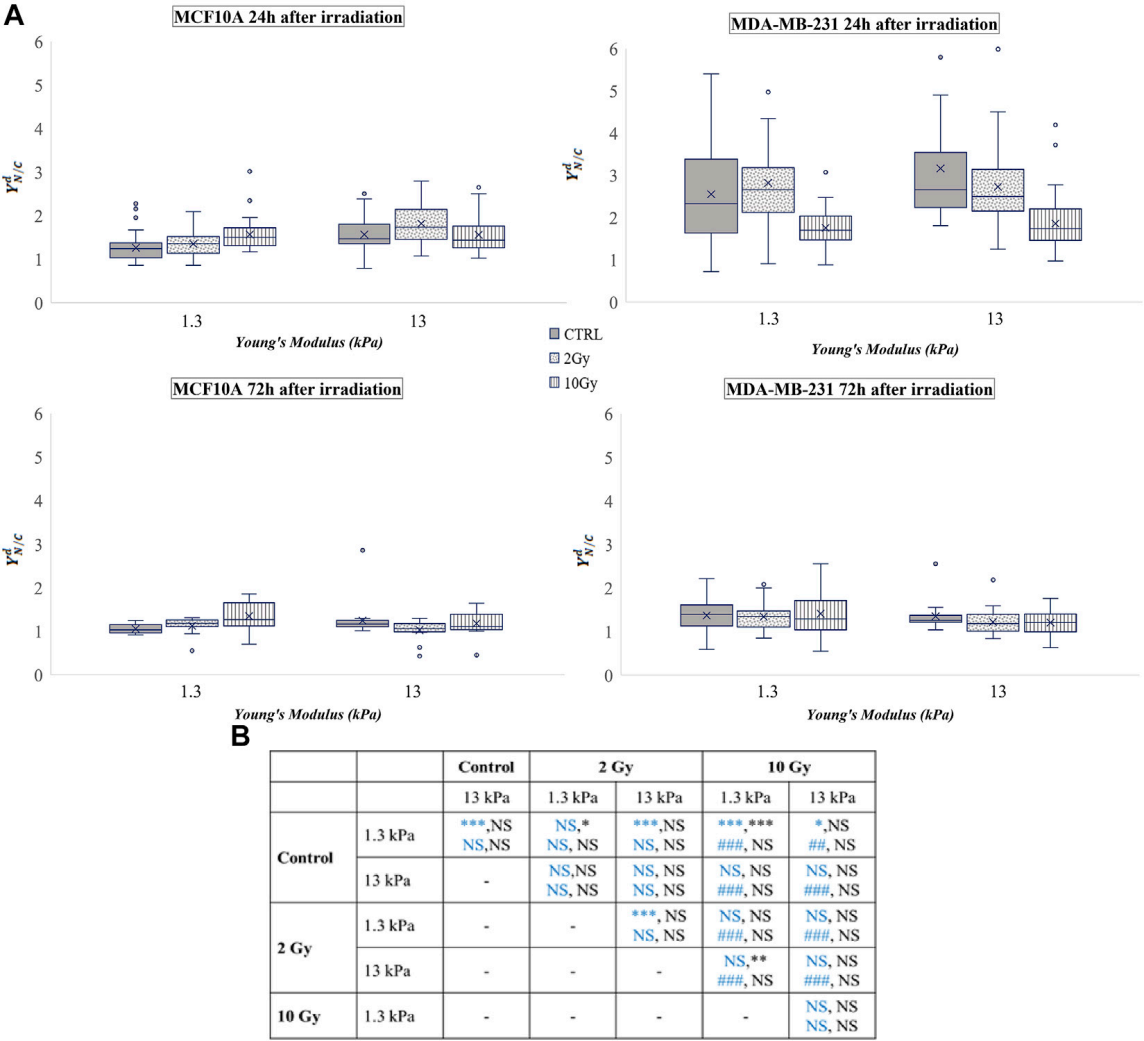
**(A)** Box plots in which the mean value, the median, the interquartile, and the outliers of the normalized YAP nucleus to cytoplasm ratio, $Y_{N/C}^{d}$ , are shown. The values have been estimated for both cell lines 24 (top) and 72 h (bottom) after irradiation. MCF10A on: 1.3 kPa substrate at 24 h *n* = 50, 39,and 40 for control, 2 Gy, 10 Gy, respectively; 1.3 kPa substrate at 72 h *n* = 20 for control, 2 Gy, 10 Gy; 13 kPa at 24 h *n* = 48, 33, and 23 for control, 2 Gy, 10 Gy, respectively; 13 kPa at 72 h *n* = 20 for control, 2 Gy, 10 Gy; MDA-MB-231 on: 1.3 kPa substrate at 24 h n= 43, 35, and 41 for control, 2 Gy, 10 Gy, respectively; 1.3 kPa substrate at 72 h *n* = 51, 54, 36 for control, 2 Gy, 10 Gy, respectively; 13 kPa at 24 h *n* = 33, 46, and 60 for control, 2 Gy, 10 Gy, respectively; 13 kPa at 72 h *n* = 18, 53, and 35 for control, 2 Gy, 10 Gy, respectively. **(B)** Statistical analysis: asterisks (*) refer to $Y_{N/C}^{d}$ at 24 h (blue) and 72 h (black) of MCF10A cell. Hash signs (#) to those of MDA-MB-231 cells. ***, ^###^ *P*< 0.001. **, ^##^ *P*< 0.01. *P< 0.05. NS not significant.

This ratio was calculated for both cell lines and the used time points were 24 and 72 h after irradiation. The obtained values are shown in Figure 2, where the box plots show the mean value, the median, the interquartile range, and the outliers. The healthy cell line was characterised by a YAP ratio close to 1 on both substrates, indicating an evenly distributed signal into the cytoplasmic and nuclear compartments. A slight but significant increase of this ratio was found passing from 1.3 to 13 Young’s modulus, indicating that MCF10A cells can perceive the different mechanical properties of their microenvironment. However, the high confluence cooperates to prevent a massive translocation into the nucleus also in those mechanical conditions where YAP activity is generally promoted ( $Y_{N/C}^{d}$ >> 1) (Dupont et al., 2011). Then, we investigated the effects of irradiation after 24 h and found a dose-dependent increase of the ratio on the soft substrate (Figure 2). On the stiffer substrate, YAP concentration of MCF10A cells showed higher values than the control condition after being irradiated with a dose of 2 Gy, while the higher dose did not affect the YAP ratio.

MDA-MB-231 cells showed a YAP ratio strongly higher than 1, indicating a substantial accumulation of YAP into the nucleus. Interestingly, the value of the ratio was not significantly varied passing from 1.3 to 13 kPa Young’s modulus. This phenomenon was already confirmed by other studies (Harvey et al., 2013; Piccolo et al., 2014) since it is proven that YAP is highly active in almost all tumour cells (Zanconato et al., 2016b). In fact, in both sparse and confluent conditions, the loss of E-cadherin-β-catenin complexes directly controls the nuclear localization of YAP in tumour cells, and specifically in MDA-MB-231 (Kim et al., 2011). After irradiation, YAP concentration decreased in a dose-related manner in both conditions, supporting the idea of a repression effect of radiation exposure on the activation of YAP signalling, as previously observed also in glioma cells (Xu et al., 2019). The values obtained from the analyses carried out 72 h after irradiation show that MDA-MB-231 cells reduced the values of $Y_{N/C}^{d}$ in all conditions, exhibiting identical ratios on both substrates and in both control and irradiated conditions.

### 3.2 Radiation effects on expression levels and activation status of YAP

The analysis of $Y_{N/C}^{d}$ gives information about the subcellular YAP concentration (predominantly nuclear or cytoplasmic) but does not provide details about nuclear and cytoplasmic YAP expression intensity. Then, the quantitative evaluation of both nuclear and cytoplasmic YAP density ( $Y_{N}^{d}$ , $Y_{C}^{d}$ ) and the overall expression of nuclear and cytoplasmic YAP ( $Y_{N}$ , $Y_{C}$ ), as indicated in the subsection 2.5, was performed.

**FIGURE 3:**
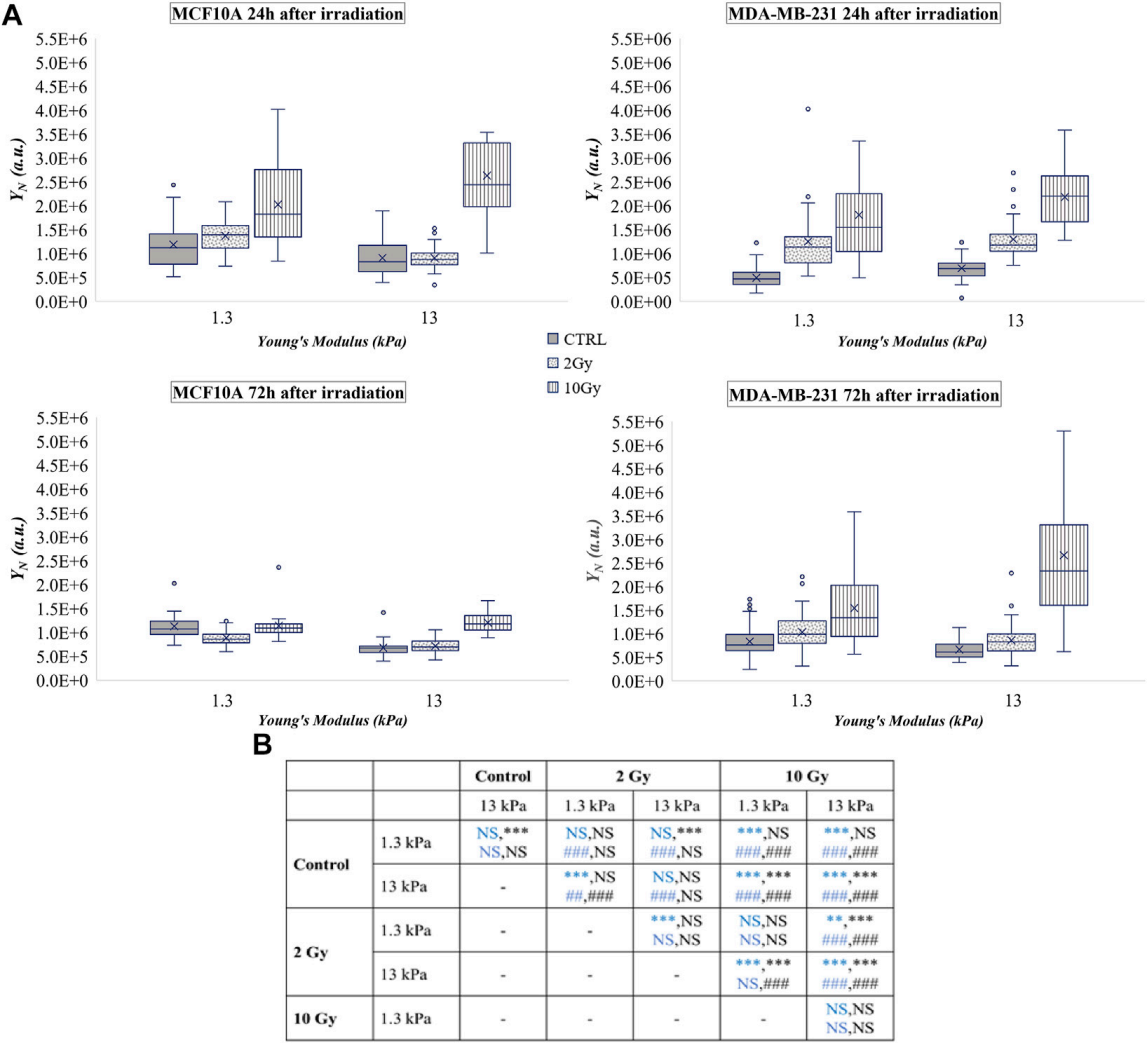
**(A)** Box plots in which the mean value, the median, the interquartile, and the outliers of the YAP expression into the nucleus, $Y_{N}$ , are shown. The values have been estimated for both cell lines 24 (top) and 72 h (bottom) after irradiation. MCF10A on: 1.3 kPa substrate at 24 h *n* = 50, 39, and 40 for control, 2 Gy, 10 Gy, respectively; 1.3 kPa substrate at 72 h *n* = 20 for control, 2 Gy, 10 Gy; 13 kPa at 24 h *n* = 48, 33, and 23 for control, 2 Gy, 10 Gy, respectively; 13 kPa at 72 h *n* = 20 for control, 2 Gy, 10 Gy; MDA-MB-231 on: 1.3 kPa substrate at 24 h n= 43, 35, 41 for control, 2 Gy, 10 Gy, respectively; 1.3 kPa substrate at 72 h *n* = 51, 54, 36 for control, 2 Gy, 10 Gy, respectively; 13 kPa at 24 h *n* = 33, 46, and 60 for control, 2 Gy, 10 Gy, respectively; 13 kPa at 72 h *n* = 18, 53, and 35 for control, 2 Gy, 10 Gy, respectively. **(B)** Statistical analysis: Asterisks (*) refer to $Y_{N}$ at 24 h (blue) and 72 h (black) of MCF10A cell. Hash signs (#) to those of MDA-MB-231 cells. ***, ^*###*^ *P* < 0.001. **, ^##^ *P* < 0.01. NS not significant.

If no dramatic effects were observed in the normalized values of N/C ratio ( $Y_{N/C}^{d}$ ), the analysis of both $Y_{N}^{d}$ and $Y_{N}$ indicates that the radiation exposure affected sensitively the healthy cells. In particular, 24 h after irradiation a slight reduction with the lower dose and a significant enhancement of $Y_{N}^{d}$ with the higher one (Supplementary Figure S3) were found, whereas $Y_{N}$ increased with both doses (Figure 3). However, all these effects were reversed or completely recovered after 72 h on the soft substrates, indicating a probable defensive role of the healthy tissue mechanical condition, as previously reported (Panzetta et al., 2020). On the other hand, cells seeded on the substrate that mimics the tumoral tissue mechanics were not affected by the lower radiation dose, while the booster dose continued to promote an accumulation process of YAP in the nucleus, even if a partial recovery was found.

The tumoral cell line showed a substantial increase of $Y_{N}^{d}$ after the delivery of both doses on both polyacrylamide substrates. If the results discussed in the previous section suggest a translocation process of YAP from the nucleus to the cytoplasm in the tumour cells, the data reported in Figure 3 and Supplementary Figure 3 clearly indicate that the analysis of the only $Y_{N/C}^{d}$ could be partial and, eventually, misleading. The enhancement of $Y_{N}^{d}$ and $Y_{N}$ observed for both doses and both stiffnesses supports, in fact, previous results indicating that the radiation exposure promotes YAP activation on various tumour cells, by impairing and increasing nuclear localization (Fernandez-L et al., 2012; Zhang et al., 2019; Zhang et al., 2021).

**FIGURE 4:**
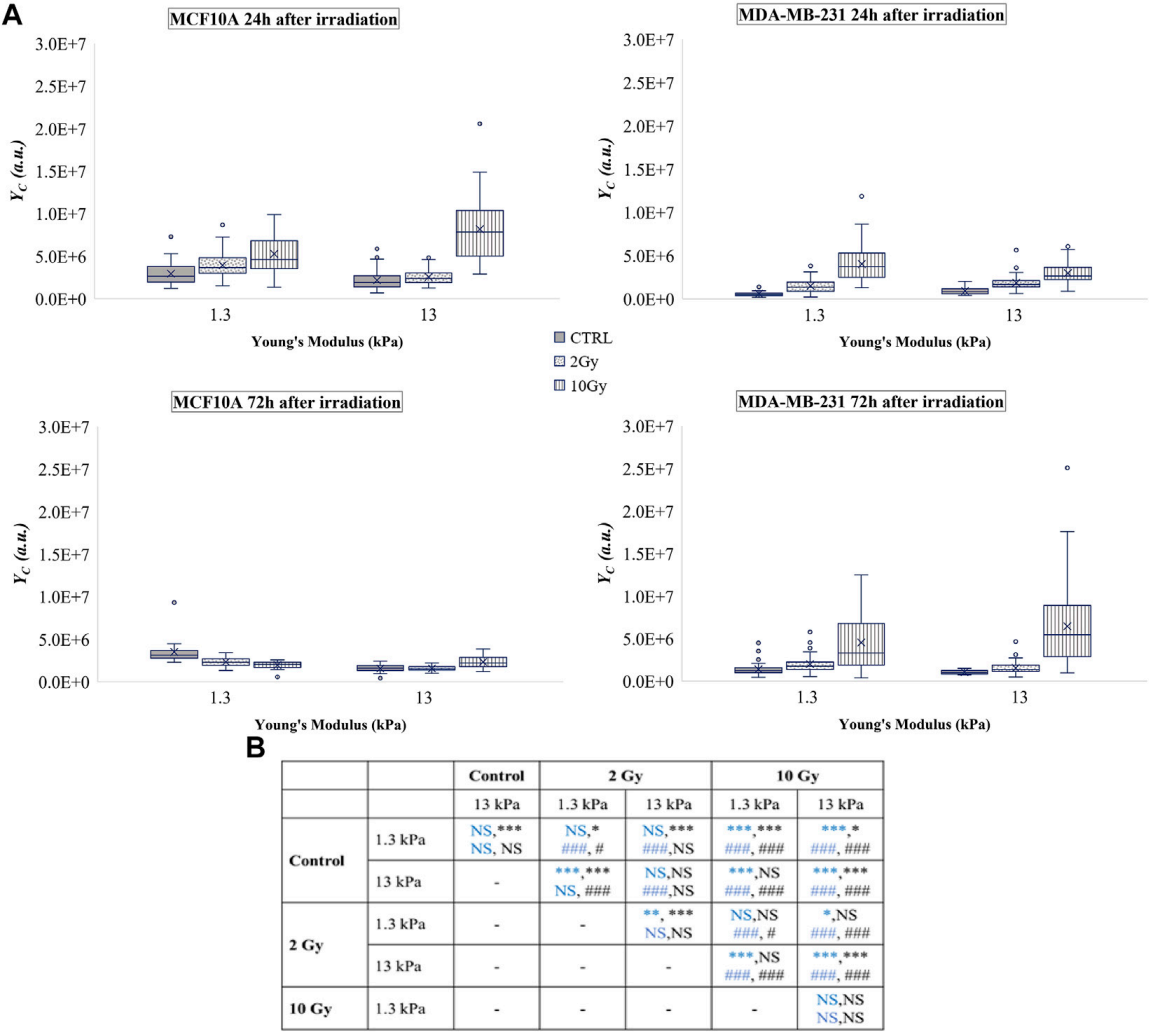
**(A)** Box plots in which the mean value, the median, the interquartile, and the outliers of the YAP expression into the cytoplasm, $Y_{C}$ , are shown. The values have been estimated for both cell lines 24 (top) and 72 h (bottom) after irradiation. MCF10A on: 1.3 kPa substrate at 24 h *n* = 50, 39, and 40 for control, 2 Gy, 10 Gy, respectively; 1.3 kPa substrate at 72 h *n* = 20 for control, 2 Gy, 10 Gy; 13 kPa at 24 h *n* = 48, 33, and 23 for control, 2 Gy, 10 Gy, respectively; 13 kPa at 72 h *n* = 20 for control, 2 Gy, 10 Gy; MDA-MB-231 on: 1.3 kPa substrate at 24 h *n*= 43, 35, and 41 for control, 2 Gy, 10 Gy, respectively; 1.3 kPa substrate at 72 h *n* = 51, 54, and 36 for control, 2 Gy, 10 Gy, respectively; 13 kPa at 24 h *n* = 33, 46, and 60 for control, 2 Gy, 10 Gy, respectively; 13 kPa at 72 h *n* = 18, 53, and 35 for control, 2 Gy, 10 Gy, respectively.**(B)** Statistical analysis: Asterisks (*) refer to $Y_{C}$ at 24 h (blue) and 72 h (black) of MCF10A cell. Hash signs (#) to those of MDA-MB-231 cells. ***, ^###^ *P* < 0.001. **P < 0.01. *, ^#^ *P* < 0.05. NS not significant. Subcellular YAP expression correlates with lamin A/C level.

At the same time, the analyses 24 h after the treatment, showed that X-rays radiation did not affect YAP concentration in the cell cytoplasm ( $Y_{C}^{d}$ ) when MCF10A cells are seeded on the softer substrate (Supplementary Figure S4), even if the overall expression of cytoplasmic YAP ( $Y_{C}$ ) increased in a manner (Figure 4). A different trend can be observed for cells seeded on the 13 kPa substrate. In fact, the delivery of the lower dose led to a significant decrease of $Y_{C}$ , while the dose of 10 Gy affected cells by increasing both $Y_{C}^{d}$ and $Y_{C}\;$ (Figure 4, and Supplementary Figure S4). 3 days after radiation, the healthy cell line showed a significant decrease of $Y_{C}^{d}$ , while on the 13 kPa substrate an opposite trend, with a dose-dependent increase in $Y_{C}$ was found.

On the other side, X-rays radiation affected tumour cells by increasing $Y_{C}^{d}$ and $Y_{C}$ on both substrates. The values resulted particularly augmented when cells were treated with the dose of 10 Gy for both time periods.

**FIGURE 5:**
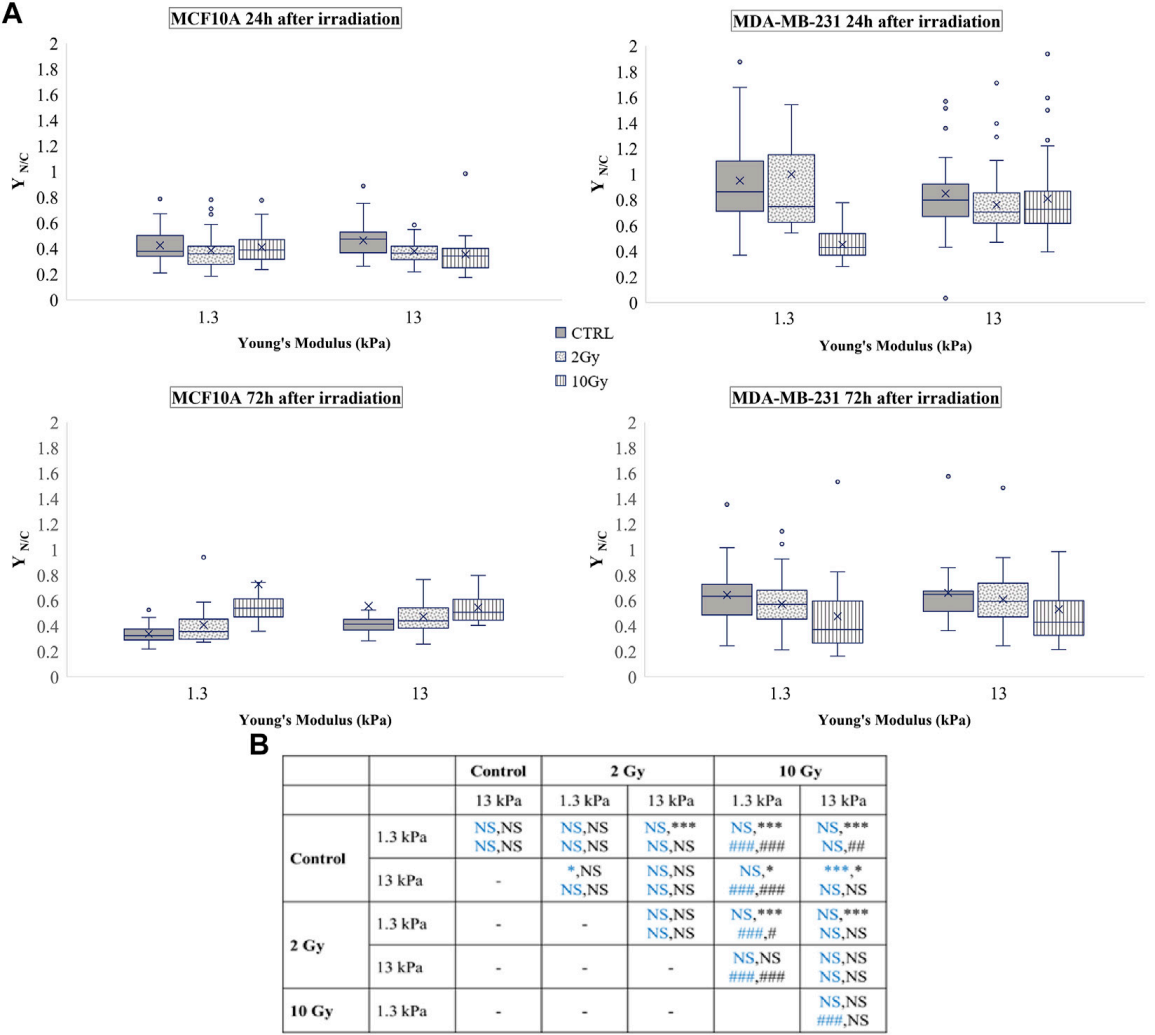
**(A)** Box plots in which the mean value, the median, the interquartile, and the outliers of the nuclear to cytoplasmic ratio of total YAP, $Y_{N/C}$ , are shown. The values have been estimated for both cell lines 24 (top) and 72 h (bottom) after irradiation. MCF10A on: 1.3 kPa substrate at 24 h *n* = 50, 39, and 40 for control, 2 Gy, 10 Gy, respectively; 1.3 kPa substrate at 72 h *n* = 20 for control, 2 Gy, 10 Gy; 13 kPa at 24 h *n* = 48, 33, and 23 for control, 2 Gy, 10 Gy, respectively; 13 kPa at 72 h *n* = 20 for control, 2 Gy, 10 Gy; MDA-MB-231 on: 1.3 kPa substrate at 24 h *n*= 43, 35, 41 for control, 2 Gy, 10 Gy, respectively; 1.3 kPa substrate at 72 h *n* = 51, 54, 36 for control, 2 Gy, 10 Gy, respectively; 13 kPa at 24 h *n* = 33, 46, and 60 for control, 2 Gy, 10 Gy, respectively; 13 kPa at 72 h *n* = 18, 53, and 35 for control, 2 Gy, 10 Gy, respectively. **(B)** Statistical analysis: Asterisks (*) refer to $Y_{N/C}$ at 24 h (blue) and 72 h (black) of MCF10A cell. Hash signs (#) to those of MDA-MB-231 cells. ***, ^###^ *P* < 0.001. ^##^ *P* < 0.01. *, ^#^ *P* < 0.05. NS not significant.

Taken together, these results indicate that, after the irradiation, the tumour cell line exhibits a profound and dose-dependent augmentation of both quote of phosphorylated ( $Y_{C}$ ) and dephosphorylated YAP ( $Y_{N}$ ). In general, it has been demonstrated that YAP silencing potentiates sensitivity of breast cancer cells to radiation therapy (Andrade et al., 2017) and that, on the contrary, the overall overexpression of YAP (here found particularly in cells irradiated with the booster dose and cultured on stiff substrates) might upregulate the expression levels of some anti-apoptosis genes, such as BCL2L1 and BIRC5, then decreasing progressively the apoptotic sensitivity of tumour cells (Lee and Yonehara 2012; Rosenbluh et al., 2012). However, as already reported, more than the whole expression level of YAP, its nucleo-cytoplasmic distribution effectively describes YAP activity regulated by upstream core components of the Hippo pathway (Piccolo et al., 2014). The coactivating transcriptional function of YAP, in fact, is restrained when the activation of the Hippo pathway produces its serine phosphorylation and the consequent cytoplasmic sequestration (Hansen et al., 2015). Taking this into account, the localization of YAP was quantified in terms of $Y_{N/C}$ in order to better define the effects of radiation on its activation status. On one hand, MDA-MB-231 maintained at 24 h the global $Y_{C}$ and $Y_{N}$ at similar values, as indicated by the unaltered value of $Y_{N/C}$ , except on the soft substrate where the higher dose induced its significant reduction (Figure 5). On the other hand, the booster dose induced a global reduction of the same parameter after 72 h, even if not in a significant way on the stiffer substrate. Similarly, the healthy cell line manifested a substantial reduction of $Y_{N/C}$ on the stiff substrate after 24 h when irradiated with the higher dose, whereas at longer time this response was completely reversed with a dose-dependent increase of the same parameter on both substrates (Figure 5).

In late response to irradiation, the process of YAP sequestering in the nucleus of MCF10A or in the cytoplasm of MDA-MB-231 could be a mechanism by which cell growth or apoptosis are regulated. Dephosphorylation of YAP, that associates with its transportation in the nucleus, has been shown to reduce p73 binding and the consequent cell apoptosis downstream in breast cancer cells (Matallanas et al., 2007). However, other researches have revealed that phosphorylation of YAP in response to ionizing radiation might impede YAP functioning as co-activator of p73 to enhance proapoptotic genes, thereby contributing to cell protection (Strano et al., 2005; Levy et al., 2008) (Lapi et al., 2008).

### 3.3 Subcellular YAP expression correlates with lamin A/C level

**FIGURE 6:**
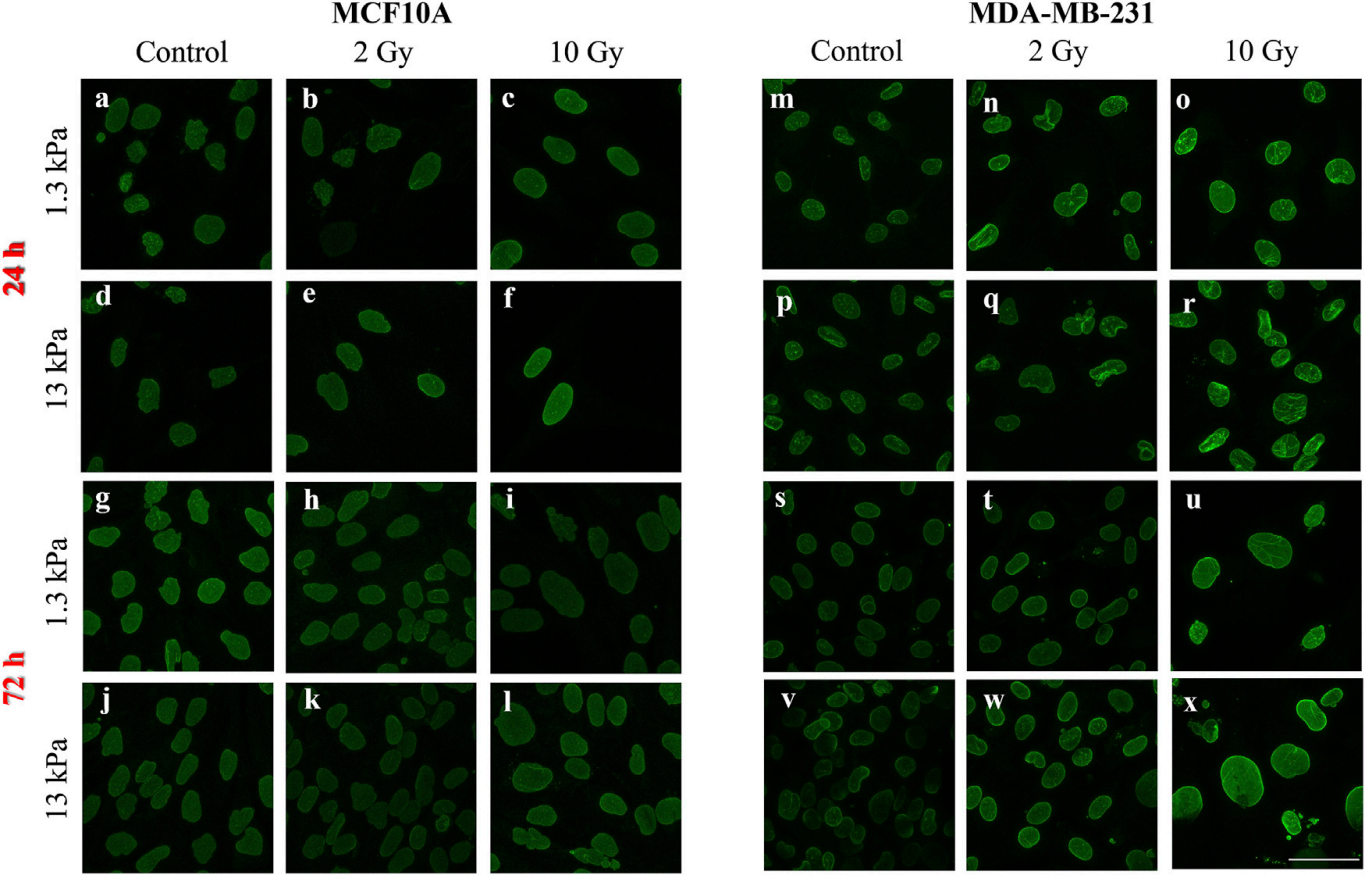
Representative images of lamin A/C immunofluorescence in MCF10A **(A–L)** and MDA-MB-231 **(M–X)** are shown. Scale bar, 50 μm.

**FIGURE 7:**
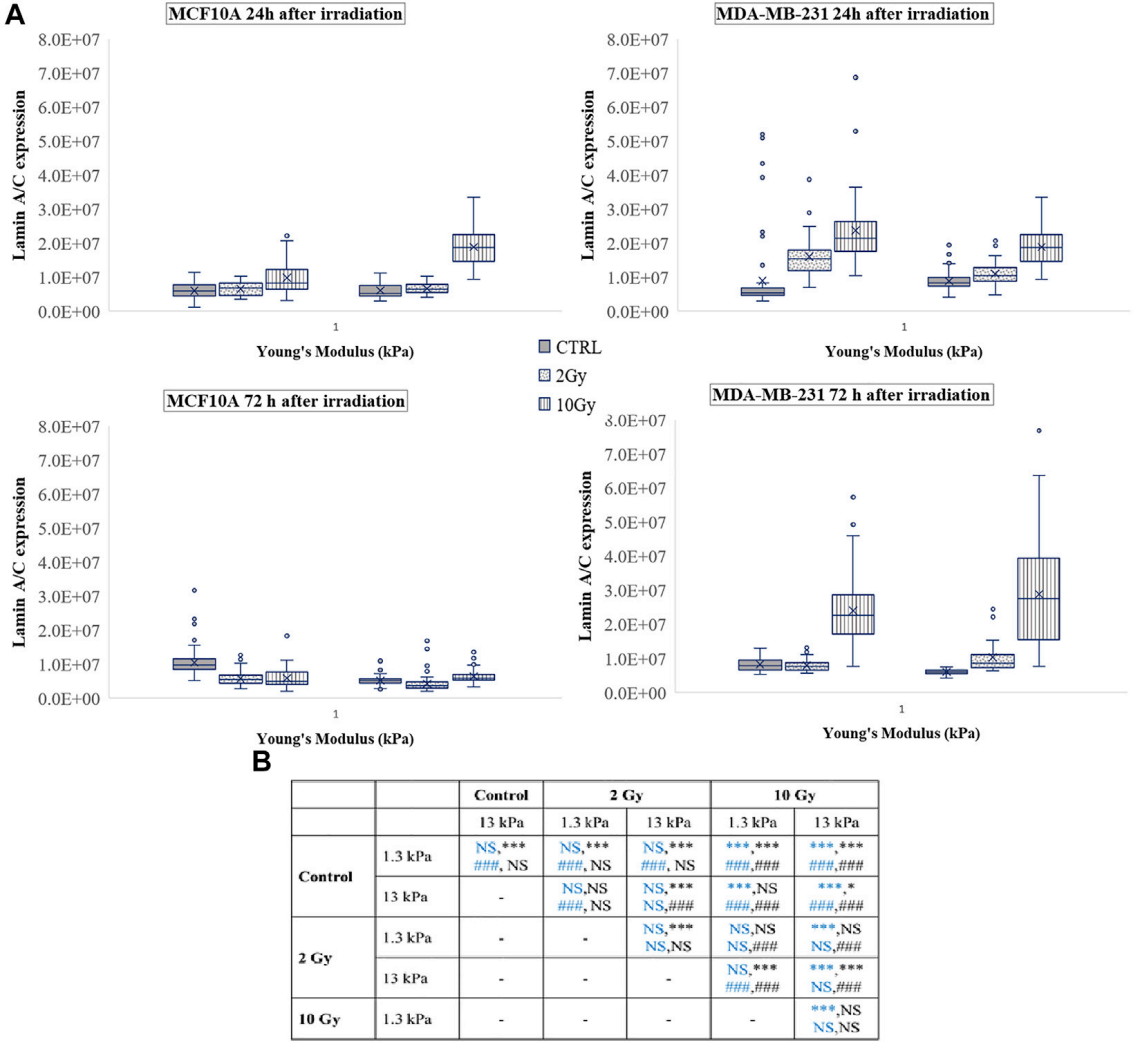
**(A)** Box plots in which the mean value, the median, the interquartile, and the outliers of the levels of lamin A/C expression are shown. The values have been estimated for both cell lines 24 (top) and 72 h (bottom) after irradiation. MCF10A on: 1.3 kPa substrate at 24 h *n* = 50, 40, and 42 for control, 2 Gy, 10 Gy, respectively; 1.3 kPa substrate at 72 h *n* = 89, 78, and 70 for control, 2 Gy, 10 Gy, respectively; 13 kPa at 24 h n = 43, 41, 23 for control, 2 Gy, 10 Gy, respectively; 13 kPa at 72 h *n* = 75, 83, and 63 for control, 2 Gy, 10 Gy, respectively; MDA-MB-231 on: 1.3 kPa substrate at 24 h *n* = 99, 42, and 33 for control, 2 Gy, 10 Gy, respectively; 1.3 kPa substrate at 72 h *n* = 22, 25, and 37 for control, 2 Gy, 10 Gy, respectively; 13 kPa at 24 h *n* = 108, 40, and 37 for control, 2 Gy, 10 Gy, respectively; 13 kPa at 72 h *n* = 22, 39, and 35 for control, 2 Gy, 10 Gy, respectively. **(B)** Statistical analysis: Asterisks (*) refer to lamin A/C at 24 h (blue) and 72 h (black) of MCF10A cell. Hash signs (#) to those of MDA-MB-231 cells. ***, ^###^ *P* < 0.001. *P < 0.05. NS not significant.

A vast literature indicates the key role of nuclear deformability in mediating changes in YAP localization (Elosegui-Artola et al., 2017; Kalukula et al., 2022; Maremonti et al., 2022). It has been demonstrated, in fact, that cells with stiffer nuclei require greater contractile forces from the cytoskeleton to compress the nucleus and to evoke YAP shuttling from the cytoplasm to the nucleus (Koushki et al., 2020). On the other side, the key role of lamin A/C in regulating nuclear stiffness (Koushki et al., 2020) led us to question if the changes in YAP localization after irradiation can be correlated to variations in lamin A/C expression level (Figure 6). As shown in Figure 7, at short time the irradiation increased in a dose-dependent manner the lamin A/C expression in both cell lines and on both stiffnesses. At longer time, this response was completely reversed in the healthy cells and accompanied by the nuclear translocation of YAP. On the contrary, the higher levels of lamin A/C, together with the reduction of the nuclear localization of YAP (Figure 5), persisted in the tumour cells, when irradiated with the booster dose. These findings suggest that the variations of YAP n/c expression ratio could be ascribed to the effects that the irradiation can have on lamin A/C levels and, consequently, on the nuclear deformability.

## 4 Conclusion

In this study, two mammary cell lines, the healthy MCF10A and the cancerous MDA-MB-231, were employed to investigate the changes in the expression of the YAP protein before and after radiation treatment. Cells were irradiated with doses used in the conventional radiotherapy treatment, 2 and 10 Gy, and analysed 24 and 72 h after the treatment. Additionally, cells were seeded on polyacrylamide substrates with two different Young’s modulus, 1.3 and 13 kPa, that emulate the healthy and tumour tissue respectively, to evaluate the role of the ECM in this process.

Our results showed that X-ray irradiation affected in a significant way the levels of YAP expression, density, and localization in both cell lines. The early short time response (24 h) results to be transient in the healthy cells; in fact, MCF10A, after an overall increase of YAP level in both the nucleus and cytoplasm and on both substrates, reduced mostly the YAP expression in the cytoplasm by inducing a translocation process into the nucleus, dependent on both substrate stiffness and X-ray dose. Tumour cells responded similarly to the healthy ones at short time, but the effects of X-ray were completely reversed at 72 h in terms of subcellular localization, as indicated by the reduction of $Y_{N/C}$ .

Since YAP works as a transcriptional co-activator, its localization into the nucleus before and after irradiation could have a different impact on subsequent cell behaviour. In particular, the reduced expression of YAP and its translocation into the nucleus could be a mechanism by which healthy cells protect themselves from apoptosis (Zhang et al., 2012) and control their growth (increased $Y_{N/C}$ associates also with growth process). On the other side, the YAP nuclear exclusion/reduction can result in a temporary confined inhibition of cell proliferation and invasion, as supported by previous findings (Panzetta et al., 2020; La Verde et al., 2021), but more importantly in a modulation of cell sensitivity to radiation (Tsujiura et al., 2014) that can be used in the design of doses and timing of subsequent radiation therapy.

These results can aid in obtaining a deeper knowledge of the role of the ECM and the effect of radiotherapy on both healthy and cancerous cells and in developing the diagnostic and therapeutical aspects of radiation therapy.

## Data Availability

The raw data supporting the conclusion of this article will be made available by the authors, without undue reservation.
