# Supplementary material for: Radiation therapy affects YAP expression and intracellular localization by modulating lamin A/C levels in breast cancer
Source: Front Bioeng Biotechnol. 2022 Aug 24;10:969004. doi: 10.3389/fbioe.2022.969004 (PMC9450017; doi:10.3389/fbioe.2022.969004)
Supplement: Supplementary file 1 [file DataSheet1.PDF]

Supplementary Material

Radiation therapy affects YAP expression and intracellular localization by modulating lamin A/C levels in breast cancer

Giuseppe La Verde[1,2], Valeria Artiola[3], Mariagabriella Pugliese[1,3], Marco La Commara[1,2], Cecilia Arrichiello[4], Paolo Muto[4], Paolo A. Netti[5,6], Sabato Fusco[7]*, Valeria Panzetta[5,6]

[1]Istituto Nazionale di Fisica Nucleare, INFN sezione di Napoli, Via Cinthia ed. 6, 80126 Naples, Italy

[2]Dipartimento di Farmacia, Università degli Studi di Napoli Federico II, Via Montesano 49, 80131 Naples, Italy

[3]Dipartimento di Fisica "Ettore Pancini", Università degli Studi di Napoli Federico II, Via Cinthia ed. 6, 80126 Naples, Italy

[4]Radiotherapy Unit, Istituto Nazionale Tumori-IRCCS-Fondazione "G. Pascale", Via Semmola, 53, 80131 Naples, Italy

[5]Interdisciplinary Research Centre on Biomaterials (CRIB) and Dipartimento di Ingegneria Chimica, dei Materiali e della Produzione Industriale, Università degli Studi di Napoli Federico II, Piazzale Tecchio 80,80125 Naples, Italy

[6]Center for Advanced Biomaterials for Healthcare@CRIB, Istituto Italiano di Tecnologia, Largo Barsanti e Matteucci 53, 80125 Naples, Italy

[7]Department of Medicine and Health Sciences "V. Tiberio", University of Molise, Campobasso, Italy

*** Correspondence:** sabato.fusco@unimol.it

Keywords: breast cancer; mechanobiology; extracellular matrix stiffness; YAP; lamin A/C; radiotherapy.

Supplementary Figures

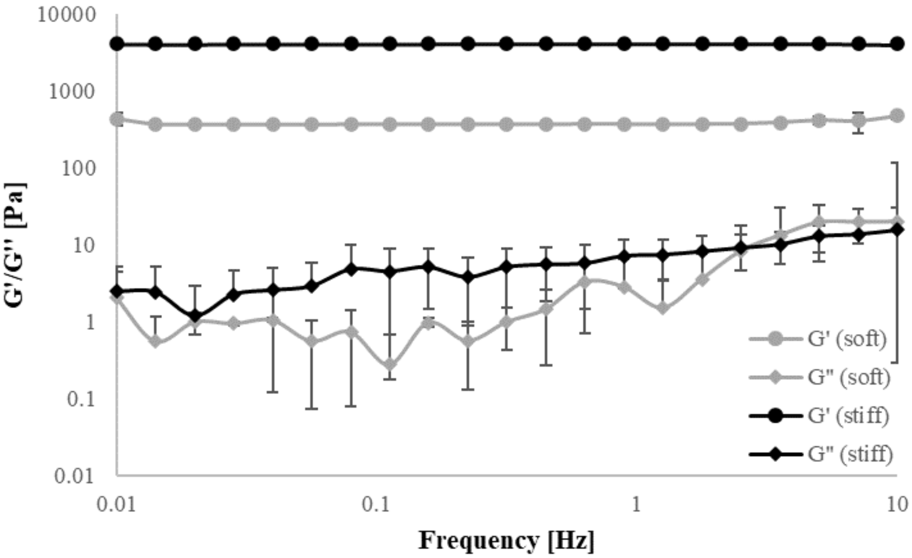

Supplementary Figure 1. Dynamic moduli, G' (circle symbols) and G'' (rhombus symbols) as a function of frequency for the two polyacrylamide formulations described in the subsection 2.1 (soft refers to formulation composed by 4% acrylamide and 0.15% methylene-bis-acrylamide, indicated as ~ 1 kPa in the manuscript, light grey curves; stiff refers to formulation composed by 10% acrylamide and 0.1% methylene-bis-acrylamide indicated as 13 kPa, black curves). Polyacrylamide presents a flat dynamic elastic modulus (G') curve and, therefore, no dependency of their elastic characteristics upon the frequency. The viscous modulus (G'') presents a slight dependence upon frequency, but its value remains sensitively lower than that of G' (G''/G'<0.1 in all the frequency range), delineating a typical elastic behaviour. The Young's modulus for the two formulations (soft refers to ~1 kPa, stiff to 13 kPa) has been determined by using the formula $2(1+\nu)G$, where ν is the Poisson's ratio, set equal to 0.457 (Takigawa et al., 1996), ang G is the complex modulus $G = G' + G''$, equal to ~0.4 kPa and ~4 kPa for soft and stiff formulations, respectively.

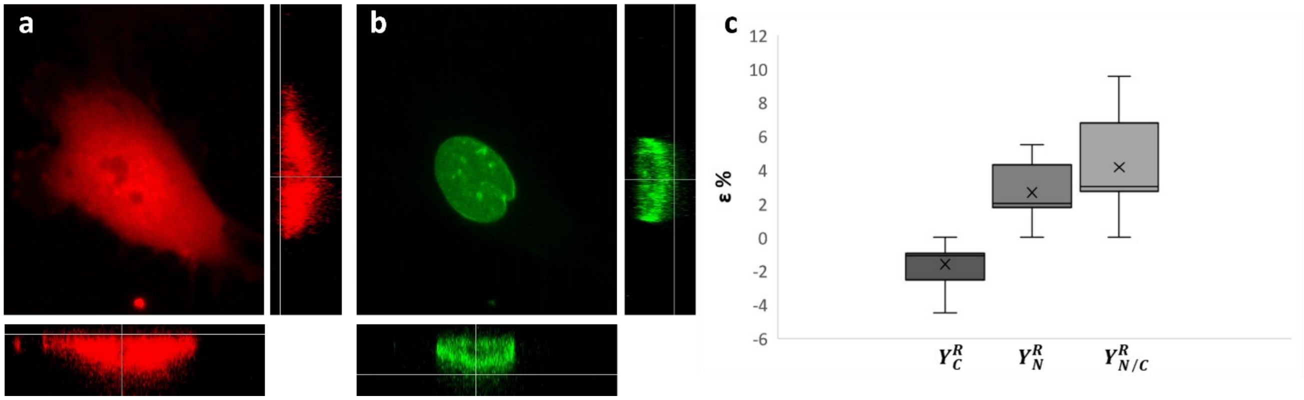

Supplementary Figure 2. Representative images of sum intensity projections of z-stack images and orthogonal views taken from YAP (a) and lamin a/c (b) immunofluorescence. The orthogonal views show that the nucleus accounts for almost the whole cell height and that, consequently, the number of slices on the top and on the bottom of the nucleus is close to zero. Following the methodology reported in the subsection 2.6, the error $\varepsilon\%$ committed for the evaluation of Y_N, Y_C and $Y_{N/C}$ was quantified for 13 cells. We found that Y_C is slightly underestimated (~1.6%), and Y_N and $Y_{N/C}$ slightly overestimated (~2.6% and ~4.2%, respectively), demonstrating that the error $\varepsilon\%$ does not affect the variations observed between the control and irradiated conditions.

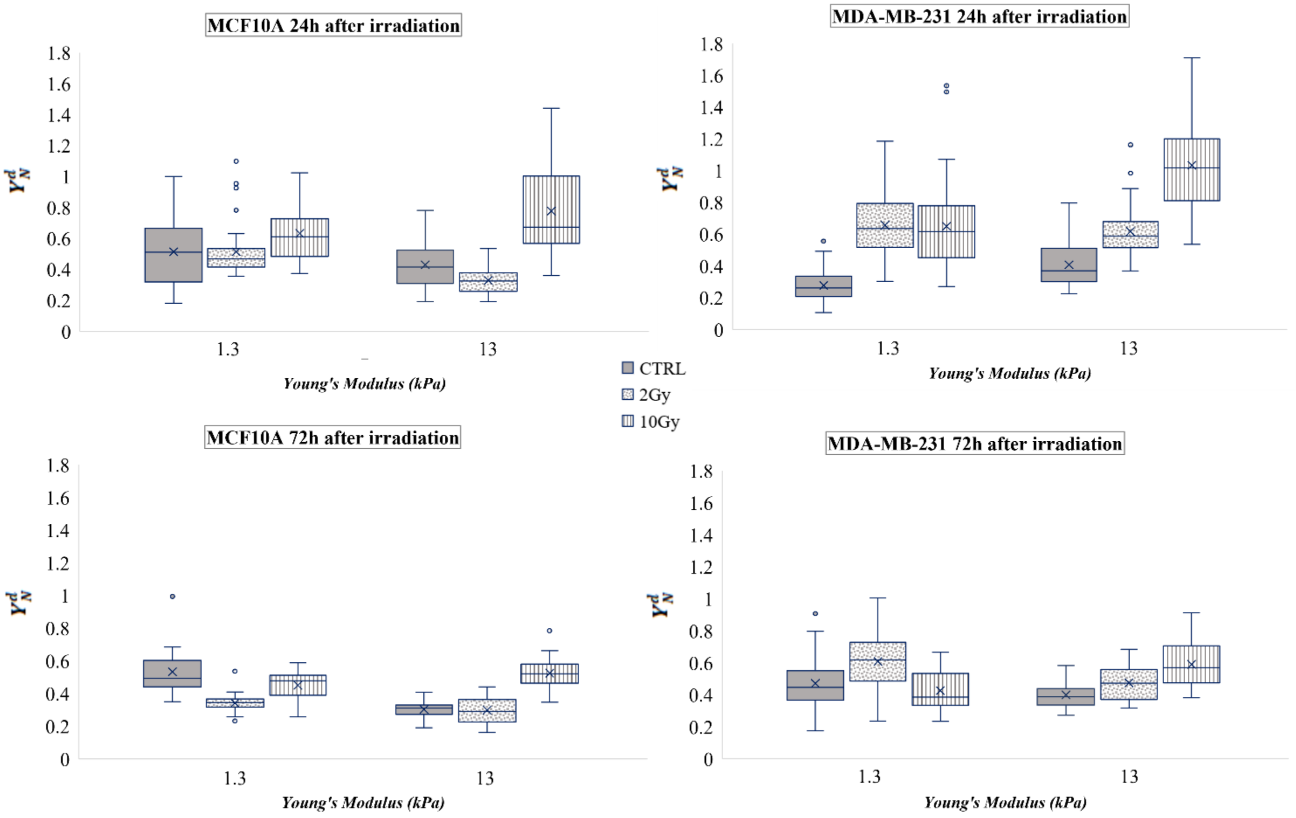

Supplementary Figure 3. Box plots of YAP Nucleus to Nucleus Area Ratio estimated for the healthy and the tumoral cell line on both substrates. The analyses have been carried out 24 (top) and 72 hours (bottom) after irradiation.

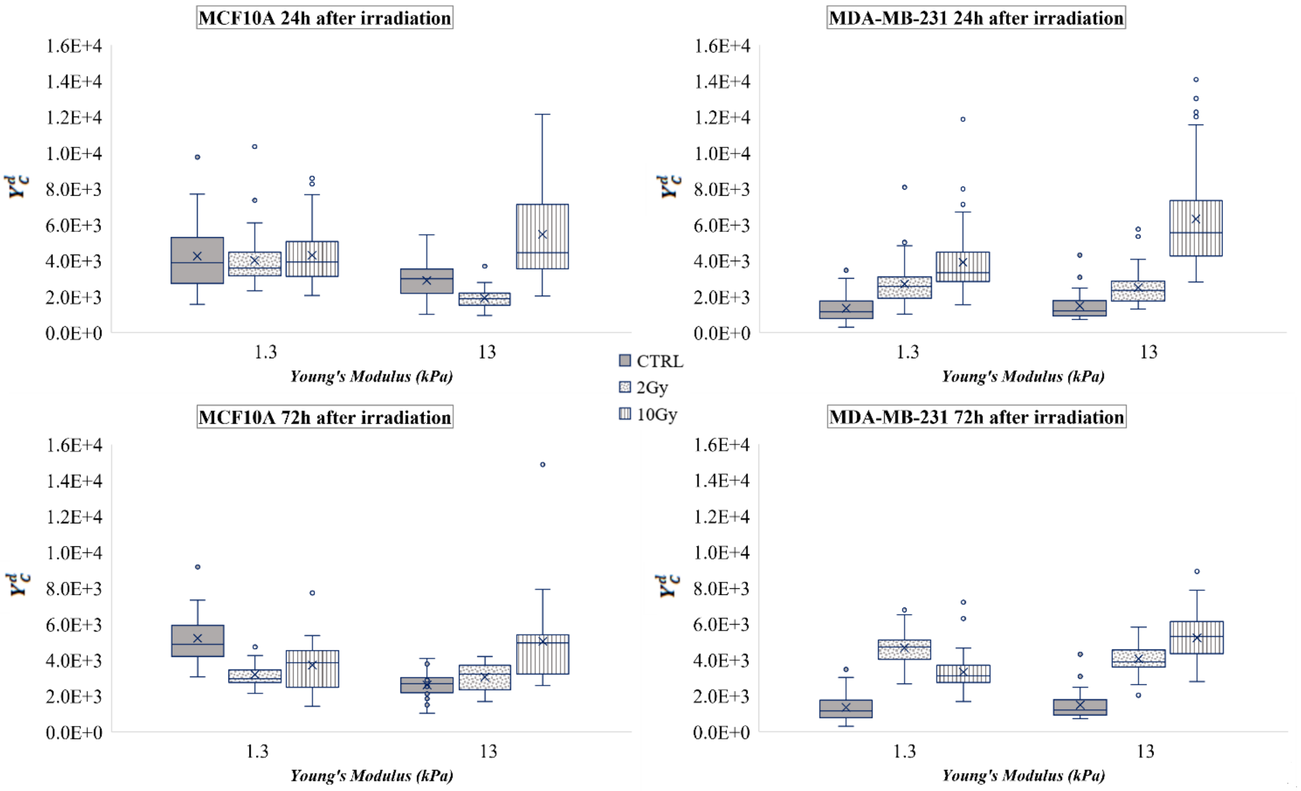

Supplementary Figure 4. The box plots of YAP Cytoplasm to Cytoplasm Area Ratio, calculated for both cell lines 24 (top) and 72 hours (bottom) after irradiation.

References

Takigawa, T., Morino, Y., Urayama, K., & Masuda, T. (1996). Poisson's ratio of polyacrylamide (PAAm) gels. Polymer Gels and Networks, 4(1), 1-5.
